# Dr. C. T. Jackson’s Remarks on the Discovery of Ethereal Inhalations

**Published:** 1847-05

**Authors:** 


*Dr. C. T. Jackson's remarks on the discovery of Ethereal inhalations.*

The following remarks were made by Dr. Jackson recently, on a public occasion, respecting his claim to the discovery of the application of the ethereal vapor to surgical operations. We quote from the Boston Med. and Surg. Journal.—Ed. Buff. Jour.

Dr. C. T. Jackson being called upon by Dr. Bigelow, said that he had little new to communicate to the gentlemen present, since they had enjoyed frequent opportunities of witnessing the effects of ethereal inhalation during the past winter, and were already aware of its power in the prevention of pain during surgical operations. He would, however, take this opportunity of vindicating his own and his country's claims to the honor of this discovery.

He was aware of the pretensions advanced by others, but he believed they had not been countenanced by the scientific world.—The Academy of Sciences of France had received this discovery, and acknowledged its value, and had recorded it as emanating from America. They have set aside, at once, the claims of pretenders, and have acted justly and honorably.

He had already given to the public an account of the original experiments which he had made, on the effects of ether vapor. He would only re-assert, as he can prove by the testimony of others, that he discovered that insensibility to pain was produced by inhalation of sulphuric ether vapor, and that he communicated the fact to one of his pupils in February, 1846, and requested him to try the experiment when he had a tooth extracted.

In the latter part of September last, he communicated this discovery to a dentist of this city, (Mr. W. T. G. Morton,) and requested him to administer the ether to one of his patients, with the assurance that it would produce insensibility, and that the experiment would be free from danger, if his directions were followed.—He regarded himself as responsible for the results of the first experiments, which were made at his suggestion, and by his advice. He next requested that dentist to go to the Massachusetts General Hospital, and ask Dr. Warren's permission to administer the ether vapor to a patient, about to undergo a surgical operation.

He regretted that any misunderstanding should have arisen concerning this discovery. He was willing to allow great credit to others for their enterprise and zeal in promoting its introduction, and for skill in improving his originally simple apparatus. He did not see any reason why each party should not be willing to rest content with what they had done.

It would certainly be unwarrantable for the miner, who carried Davy's safety lamp into the fire damps of a mine, to dispute the claims of its original inventor; for he received that instrument already proved to be efficient, with the assurance that it would guide him in safety amid the explosive gases of the mine.

